# 

**Published:** 1847-10

**Authors:** 


### *Ohio College of Dental Surgery.*

This Institution, which has been in operation for the last two years, is centrally situated in this city, and was especially erected for the purpose of a Dental College—having reference to the facilities of an Anatomical, Chemical, Mechanical and Operative Departments—besides a general Lecture Room, arranged with elevated seats, and sufficiently large to comfortably accommodate over one hundred students.

The Course of Instruction is by Oral Lecture and by Demonstration. Every operation in Mechanical as well as Operative Dentistry is particularly demonstrated before the class, and they in turn expected to perform the same. Every opportunity is thus afforded for the acquisition of practical knowledge, and to further aid in this object, the services of Mr. Hunter have been secured as Demonstrator of Mechanical Dentistry—who will take charge of the Labaratory of the Institution, and illustrate all the Manipulations of the Art, including the Manufacture of Block Teeth, for whole or partial setts.

But the principles of our Science are not neglected, for the course on Anatomy is very complete, particularly the Anatomy of the Teeth—the Buccal Cavity—the Glandular, the Nervous and Arterial system—in addition to which, opportunities to each student are afforded for dissection. The Pathology of disease—with the Therapeutical treatment—is taught, as it affects not only the Dental organs, but through them the entire system, and *vice verse.*

The third course commences the first Monday of November, 1847, and although at this time but little can be positively known as to the size of the class for the approaching session, yet the Faculty feel assured, from various letters received, that at no time has the Institution been in a more flourishing condition. The course continues till the last of February, and the entire tickets cost $105—Graduating Ticket, $25. Two courses of Lectures—the last of which must be in the Institution—the other may be in a regular Medical School—will entitle the student to come forward as a candidate for graduation. Four years' practice is considered equivalent to one course of Lectures.

We have thought thus much was of sufficient importance to the Profession to secure a place in the Dental Register.—CIN. ED.

